# Why is it so difficult to implement a longitudinal clinical reasoning curriculum? A multicenter interview study on the barriers perceived by European health professions educators

**DOI:** 10.1186/s12909-021-02960-w

**Published:** 2021-11-12

**Authors:** Małgorzata Sudacka, Martin Adler, Steven J. Durning, Samuel Edelbring, Ada Frankowska, Daniel Hartmann, Inga Hege, Sören Huwendiek, Monika Sobočan, Nils Thiessen, Felicitas L. Wagner, Andrzej A. Kononowicz

**Affiliations:** 1 Department of Medical Education, Jagiellonian University Medical College, Krakow, Poland; 2 Instruct gGmbH, Munich, Germany; 3 Center for Health Professions Education, Uniformed Services University of the Health Sciences, Bethesda, MD USA; 4 Learning and Professional Development Group, School of Health Sciences, Örebro University, Örebro, Sweden; 5 Department of Bioinformatics and Telemedicine, Faculty of Medicine, Jagiellonian University Medical College, Krakow, Poland; 6 Medical Education Sciences, Medical Faculty, University of Augsburg, Augsburg, Germany; 7 Institute for Medical Education, Klinikum der Ludwig-Maximilians-Universität, Munich, Germany; 8 Institute for Medical Education, University of Bern, Bern, Switzerland; 9 Centre for Medical Education, Faculty of Medicine, University of Maribor, Maribor, Slovenia; 10 Division of Gynecology and Perinatology, University Medical Centre Maribor, Maribor, Slovenia; 11 EDU - a degree smarter, Digital Education Holdings Ltd., Kalkara, Malta

**Keywords:** Clinical reasoning, Teaching clinical reasoning, Barriers, Health professions education, Interview study

## Abstract

**Background:**

Effective clinical reasoning is a core competency of health professionals that is necessary to assure patients’ safety. Unfortunately, adoption of longitudinal clinical reasoning curricula is still infrequent. This study explores the barriers that hinder the explicit teaching of clinical reasoning from a new international perspective.

**Methods:**

The context of this study was a European project whose aim is to develop a longitudinal clinical reasoning curriculum. We collected data in semi-structured interviews with responders from several European countries who represent various health professions and have different backgrounds, roles and experience. We performed a qualitative content analysis of the gathered data and constructed a coding frame using a combined deductive/inductive approach. The identified themes were validated by parallel coding and in group discussions among project members.

**Results:**

A total of 29 respondents from five European countries participated in the interviews; the majority of them represent medicine and nursing sciences. We grouped the identified barriers into eight general themes: Time, Culture, Motivation, Clinical Reasoning as a Concept, Teaching, Assessment, Infrastructure and Others. Subthemes included issues with discussing errors and providing feedback, awareness of clinical reasoning teaching methods, and tensions between the groups of professionals involved.

**Conclusions:**

This study provides an in-depth analysis of the barriers that hinder the teaching of explicit clinical reasoning. The opinions are presented from the perspective of several European higher education institutions. The identified barriers are complex and should be treated holistically due to the many interconnections between the identified barriers. Progress in implementation is hampered by the presence of reciprocal causal chains that aggravate this situation. Further research could investigate the perceptual differences between health professions regarding the barriers to clinical reasoning. The collected insights on the complexity and diversity of these barriers will help when rolling out a long-term agenda for overcoming the factors that inhibit the implementation of clinical reasoning curricula.

**Supplementary Information:**

The online version contains supplementary material available at 10.1186/s12909-021-02960-w.

## Background

The growing rate of medical errors is a challenge for global health care [1]. Many errors are believed to result from flaws at different stages of the clinical reasoning process [2]. Clinical reasoning (CR) is a complex process that uses cognition and discipline-specific knowledge to gather and analyze patient information, evaluate its significance, and weigh alternative actions [3]. It includes tasks such as data gathering and interpretation, synthesizing information, generating hypotheses and diagnoses, developing management plans, and avoiding cognitive errors [4]. Many experts in medical education include clinical reasoning as a core professional competency for practicing clinicians [5].

The literature suggests that explicit teaching of clinical reasoning must start from the very beginning of medical school [6]. Moreover, studies suggest it should be taught longitudinally (i.e. developed and assessed at several points throughout the curriculum) [5, 7]. Although effective clinical reasoning is believed to be central to being a health professional, previous studies have shown that only one in four medical schools actually have courses that explicitly teach clinical reasoning [8, 9]. What is more, studies suggest that existing initiatives are uncoordinated and often still in the development phase [10]. Survey studies also reveal limited faculty development opportunities in clinical reasoning teaching despite the clear demand [8, 9].

While prior studies have attempted to summarize the barriers that hinder the implementation of clinical reasoning teaching in health professions curricula, they were based primarily on experts’ opinions [5, 6] or were limited to quantitative surveys [8, 9]. However, this prior work does not allow an in-depth analysis of the causes for the low adoption levels of clinical reasoning teaching. Furthermore, many of the studies focused on the North American perspective [5, 8], which may expose factors that are not the same as those that are important for the European model of health professions education.

Therefore, the aim of this study was to explore the barriers hindering clinical reasoning teaching in the European context and to investigate the perspectives of diverse health professionals from a variety of educational roles. We do not limit our scope of interest to student curricula, but we also explore faculty development programs as an integral part of the clinical reasoning education process. We attempt to determine the perceived reasons that explain the low levels of adoption of longitudinal clinical reasoning curricula at medical and health professions schools.

## Methods

### Study design

We designed a qualitative study to explore barriers to the implementation of clinical reasoning curricula in Europe. Our research design fits into the interpretivist worldview [11]. Data were collected in semi-structured interviews held with respondents recruited by six European medical and health professions schools. We performed a thematic analysis of the collected data (Qualitative Content Analysis (QCA) variant, as described by Schreier [12]. The study was exempted from a detailed ethical review by the Institutional Review Board of the University of Bern, Switzerland (decision Req-2020-00074).

### Context

The context for the investigation was the European DID-ACT project (“Developing, implementing, and disseminating an adaptive clinical reasoning curriculum for healthcare students and educators”) in which the authors participate. The project is funded by the European Commission Erasmus+ grant framework [13]. This three-year project, which runs from 2020 to 2022, aims to design a longitudinal clinical reasoning curriculum. Higher-education institutions from six European countries (Germany, Malta, Poland, Slovenia, Sweden, Switzerland) are involved and are supported by associated partners across the globe. The DID-ACT project unites a balanced mix of medical and health professions schools with long traditions (Jagiellonian University in Kraków, University of Bern) and a relatively new one (Augsburg University, Örebro University, and Maribor University). The sixth partner is EDU, which is a Malta-based virtual medical higher education institution that employs staff across Europe [EDU].

### Participants

Participants in the study were invited by the DID-ACT partners using a purposive sampling approach [14]. Each partner recruited stakeholders from a diverse range of health professions (physicians, nurses, physiotherapists, occupational therapists, veterinarians) with diverse educational roles (tutors, course directors/coordinators, curriculum designers) and with a mixed range of experience (junior to senior educators). We chose this approach as our intention was to represent a typical snapshot of those involved in the planning and teaching of learning activities that have a focus on clinical reasoning instruction.

### Data collection

Prior to the study, we aimed to do 3–5 interviews at each academic partner institution to maintain a balance of where the opinions came from; also, checking for thematic saturation gave us a way to signal the need to recruit more participants. This divided the data collection process into two phases. During the first phase, we conducted 24 interviews followed by a preliminary summary of the data. For the second phase, we added five more interviews to check whether thematic saturation had been reached.

We developed an interview guide based on the findings of our former study that reported on the results from an on-line survey into the worldwide adoption of clinical reasoning curricula [9] and discussions among study authors from multiple European nations.

After introducing the interviewees to the project, we collected information about their professional experience and then conducted the interviews using two guiding questions: “What, in your opinion, are the main barriers/challenges to introducing a longitudinal curriculum on clinical reasoning at your institution?” and “What critical aspects/barriers/challenges do you see in implementing a train-the-trainer [clinical reasoning teaching] course at your institution?”. After the conversational element of the interviews had naturally ceased, the interviewees were then presented with a printout containing a list of the barriers we identified in our former study [9]. This list provided an opportunity for the interviewers to initiate a discussion concerning important topics that were not brought up during the interviews. In some cases, this delivered additional insights into the interviewees’ perceived barriers.

The study was conducted over 3 months: March–May 2020. Each interview was held in the native language of the respondents (German, Polish, Slovenian, Swedish) and was conducted by one of the following authors involved in the study: DH, FW, MA, MSo, MSu, NT, and SE. The interviews were conducted remotely either via phone or by teleconference and were audio-recorded after obtaining informed consent from each participant. The identity of the respondents was encoded to retain confidentiality. The interviews were transcribed and translated into English for further analysis.

### Analysis

We performed a qualitative content analysis following the methodological guidance of Schreier [12] and Gibbs [15]. After collecting the main planned series of 24 interviews, we constructed a coding frame using a combined deductive/inductive approach [12]. In the first phase, three experienced researchers (AK, MA, MSu) individually assigned the identified barriers to the coding frame themes using a concept-driven frame taken from our former study [9]. Because many of the segments were difficult to assign using the existing coding frame we decided to modify it using a data-driven strategy. In the next phase, we re-coded all the material using the new coding frame in the same team of researchers (MSu, MA, AK) discussing the differences, and refining the definitions in the coding frame until agreement was reached. We reached thematic saturation by conducting and coding five more interviews which did not introduce new themes. Following Schreier’s recommendations [12], the final coding frame was verified for content validity by the following researchers, who were not involved in constructing the coding frame but are experienced in clinical reasoning research: FW, IH, NT, SD, SE, and SH. The verification process involved both an offline review and an online discussion concerning the collected data and proposed coding frame. This led to the final version of the coding frame. Also discussed in this group were the implications of the collected data for the immediate follow up steps aimed at reducing the barriers. The consistency of coding (reliability) was checked by re-coding the segments using the final version of the coding frame by two authors (MSu, AK).

## Results

**Fig. 1:**
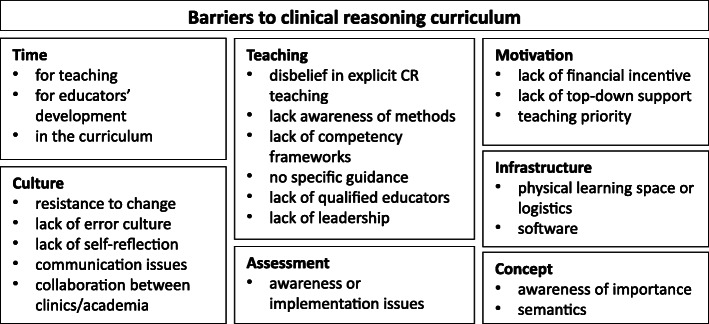
Main themes and subthemes around the barriers that hinder the adoption of a longitudinal clinical reasoning curriculum

A total of 29 respondents (17 physicians, seven nurses, three physiotherapists, two veterinary medicine doctors and one occupational therapist) from five European countries participated in this study. The participants had an average of 15 years’ experience in health professions education. After analyzing all the collected segments, we grouped them into eight general themes (Table 1). Each theme was further split into subthemes (definitions and exemplar quotations are in Additional file 1). Figure 1 presents a graphical overview of the identified themes and subthemes.

**Table 1:** Definitions of themes in the coding frame

Theme code	Theme definition
Time	Lack of time for teaching or for learning how to teach effectively, or lack of time for teaching CR in the curriculum.
Culture	Culture-related barriers arising from established practices within the university or national health care systems, collaboration issues arising from the lack of practice in discussing errors, reflection, providing feedback, and intra−/inter-professional communication.
Motivation	A theme that includes a lack of financial (i.e. resources to pay teachers) or other forms of motivation or incentives (i.e. lack of support from authorities, low priority for teaching).
Concept	Lack of awareness of the importance of CR, and/or a disagreement of what it means within one profession or interprofessionally.
Teaching	A theme that includes a lack of: awareness or belief in the effectiveness of explicit CR teaching methods; CR competency frameworks; guidelines on how to teach CR; adequately trained instructors and leadership in implementing and running CR courses.
Assessment	Unawareness or inability to implement clinical reasoning assessment methods.
Infrastructure	Difficulties in organizing clinical reasoning teaching and assessment due to lack of physical space, adequate hardware/software infrastructure, and workflows.
Others	A residual theme containing segments we could not assign otherwise.

*Note: Definitions of subthemes and exemplar quotations can be found in Additional file* *1*

### Teaching process

The teaching process was the theme with the greatest number of assigned segments about barriers (Fig. 1). A common encountered subtheme was the lack of awareness that CR can be taught by teachers who were not aware of specific methods that could influence the process of teaching CR.

*Most people don't know how to teach clinical reasoning. There is ignorance. What you don't know, you can't implement.(I10)*

In extreme cases, some of the respondents even verged on believing that teaching clinical reasoning does not make sense at all.

*There are people who without learning CR are somehow perfectly able to reason and they can say: after all, I've learned to reason very well, I'm great at it, so why do they need to come here?(I29)*

The interviewees felt that there was a lack of guidance and detailed instructions concerning how to apply specific CR teaching.

*Additionally, there are no guidelines on how to teach CR to teachers and how to create a curriculum. Other obstacles, such as a lack of trained individuals, can be overcome, but most important is the mentality and direction behind doing it [CR teaching].(I26)*

Another perceived barrier was the lack of guides on standard learning objectives for clinical reasoning.

*Our curriculum is based on learning objectives that are provided to us by the ministry [ … ] I'm not sure, but it seems to me that there is no clinical reasoning there at all.(I7)*

Another perceived barrier was the lack of skillful constructive feedback, which should be a routine part of health professionals everyday work. Teachers often teach in the way they were taught and are not aware of the benefits of reflection or do not take the time to discuss and learn from past events.

*[A belief] wide spread especially around traditionally teachers [is]: students learn best by watching me, but CR is internal so cannot be watched.(I4)*

It should be stressed that some of our respondents commented that one reason for the low-adoption rates of the CR curricula is the lack of availability of local leaders able to implement this teaching.

*We believe we have or strive for through progression within the programs, the challenge is that many are involved and it easily becomes cluttered and unclear who has the mandate to actually take control and make decisions. That makes it hard, but I don't see how it could be any different.*(I14)

Last but not least, many commented that there was a lack of a qualified faculty to teach clinical reasoning.

*Most colleagues, including me, do not have theoretical backgrounds in CR. If we had that, our teaching would probably be better.(I25)*

### Culture-related barriers

The second important theme was related to “culture” (Fig. 1). A popular subtheme related to this theme is “resistance to change”. In some cases, this anxiety was rationalized by a perceived lack of evidence to support the change.

*Resistance can also come from not being 100% sure about if the method works or not: (...) this is an approach that has its rationale in medicine, right? (...) because first of all: do no harm.*(*I8)*

The “not invented here syndrome” was also part of this subtheme, i.e. a tendency for people to avoid investing time in changing practices that they didn’t create themselves.

*If the curriculum is too general, if the plan isn’t coherent with the [country] context, it could be difficult to practice in universities that lack, for example, physicians. [There are] international differences*.(I15)

The “lack of error culture” subtheme encompassed barriers arising when participants felt there was no culture to encourage them to learn from, analyze, and accept errors.

*If a mistake is made, in order for us to feel good, it is often, much better if we say to ourselves: don't worry it's happened, I don't know... stupid patient or it's an accident at work...(...)...or... or... if I was in your position I would have definitely made the same mistake, right? And somehow... it's not about feeling guilty and punishing yourself, but it is worthwhile looking at something again and saying: ok, what went wrong here? Where was the mistake? instead of looking away, right?* (I8)

Some participants also implied that junior staff members are afraid of questioning the behavior of their superiors. On the other hand, experienced staff members also commented on their unwillingness to express how they were thinking because this may reveal their errors and, by doing so, undermine their authority.

*Our cultural background means that we live in an environment in which we are not criticized by society, and everyone may think that individual health professionals do good clinical work. Therefore, they do not see the need for this teaching. However, it is quite difficult to know when someone reaches the appropriate ability in CR. (I25)*

We identified a subtheme in the culture-related theme that related to difficulties in communication and collaboration within CR both on an intra- and interprofessional basis and also with patients.

*It is important to realize that medicine isn’t superior [to nursing or rehab aspects].(I17)*

Finally, we identified tensions between teachers working in clinics and academia-based teachers.

### Content (CR as a concept)

Another notable theme of the perceived barriers is the lack of awareness of health professions educators of CR’s theoretical background and its importance in both academic and clinical environments.

*98% of the teachers do not know anything about CR.(I6)*

According to the feedback from the interviews, teachers use clinical reasoning intuitively and do not recognize it as a separate skill; they are surprised when they realize how complex it is.

*When your project started, my neurons somehow connected. Somehow it just didn't connect in my head before. I mean, like, it's not just a problem, I feel like... it's not an easy concept to understand, you know, clinical reasoning.(I29)*

Disagreement about the meaning of CR was another barrier emphasized by some of the participants.

*Clinical reasoning is not only about identifying and treating something, but it is much more. Like the bio-psycho-social model. We always talk about patients but we also have healthy people who come for check-ups, vaccinations, or whatever. A holistic approach for all patient groups including all religions, languages, regions, disabled persons, men/women/kids (...) I would like to see the human being as the focus. The term clinical reasoning is misleading, because the term ‘human being’ is not present, it sounds very technical. And I also miss the ambulatory context.(I20)*

### Time-related

Respondents also highlighted several barriers concerning time deficiency. These can be divided into those related to lack of teaching time, lack of curricula time, and lack of time to participate in teacher training.

Lack of time for teaching is mainly justified by clinical duties.

*They [teachers] have to deal with a heavy clinical workload which discourages them from doing something new or innovative.(I10)*

It was emphasized that curricula are already overloaded:

*There is a battle for time to teach; Once people realize that they may lose time for other things, it may be difficult. If something new is added, something else may no longer be possible.(I3)*

Clinical priorities are also given as a reason for lack of engagement in teacher training; this leads to a lack of exploring new teaching methods.

*... lack of time and clinically engaged physicians (...) difficulty in seeing the benefit. You always have a crude evaluation of the actual benefits of attending courses, sometimes you attend courses in relation to your clinical expertise but could have difficulty in seeing a concrete benefit of this kind of course.(I19)*

### Motivation/incentive

The next identified theme relates to lack of motivation and incentive. Some respondents commented on teachers’ lack of available financial resources with which to organize courses for trainers and administrative staff. It was also suggested that lack of effective promotion of curricula or teacher training could be responsible for the low motivation to adopt CR curricula.

*People have to be made aware that this course exists. It has to be communicated so that the target group receives the information.* (I1)

The final important aspect regarding motivation, is lack of support from university authorities.

*...lack of conviction that this is important among decision makers [ … ] and this is quite important, because in order to have support in implementing something new, we must have support not only from the bottom-up, but also among those who make decisions at the top.(I7)*

### Assessment of clinical reasoning

An additional theme concerned clinical reasoning assessment. Some respondents saw a problem with the lack of awareness of what constitutes good assessment methods.

*How do you know how good you are or when you have learnt enough?(I18)*

### Logistics and infrastructure

In our interviews we heard comments regarding limitations of existing technical infrastructure (including software environments) and logistical difficulties.

*To maximize the learning outcome, the group size, technical infrastructure and room availability are relevant, but these are limited. It is trivial but still important. (I23)*

### Other

We identified a few segments that could not be assigned to the previously mentioned themes and were therefore classified as “others”. Among these were comments claiming there were no barriers.

*If such a program would be offered at my institution, I would register immediately. Somehow I would make time for that. But this is only my perspective. (I23)*

## Discussion

The aim of our study was to shed more light on the barriers behind the low adoption of longitudinal clinical reasoning curricula in Europe from the viewpoint of those who provide clinical reasoning instruction. From the interviews conducted at several health professions schools, we gained insights that paint a broad picture of what may cause resistance against explicit clinical reasoning teaching. The highlighted issues related directly to the teaching of clinical reasoning, such as controversies around its meaning and how to best teach/assess it. This resistance is also explained by higher clinical priorities, a shortage of financial/infrastructural resources and cultural barriers. The latter includes resistance to change, a lack of established practices in learning from mistakes, encouraging feedback on the clinical reasoning thinking process or treatment, and tensions between health professions.

**Fig. 2:**
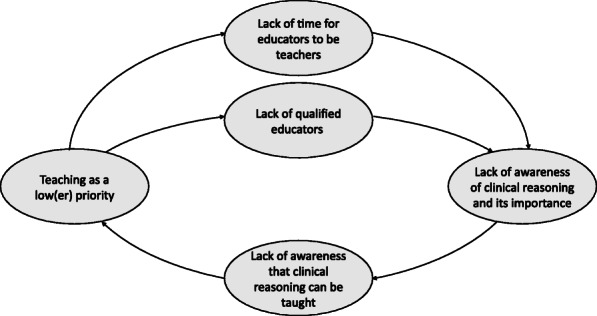
A vicious circle blocking implementation of clinical reasoning in curricula

While interpreting the obtained results, we had three major reflections. First, the barriers that hinder CR teaching should be analyzed holistically. Even if we can group them into separate themes, as was done in previous reports [8, 9], they do not exist in a vacuum and can form causative sequences and stages. For example, if you are not convinced of the existence of CR as a distinct competence or ability, or you do not consider it important in preventing medical errors, you are unlikely to be aware that it can be explicitly taught and you are probably not interested in how to teach CR most efficiently. Moreover, if you are in a community in which there is no time to reflect on performance or there is no culture of doing so, you may not regularly think about reasoning processes and how you reach the right or wrong conclusions. If you do not consider this reflection important, you do not search for or follow guidelines on how to use them in educational activities. Causative sequences may even turn into a vicious circle that blocks any progress, as illustrated in Fig. 2.

Second, the theme that in our view had a major impact and therefore could be regarded as a central barrier is the lack of educators qualified in teaching clinical reasoning. As already observed, e.g. by Cuhna et al., clinical reasoning is best learned from an inspired teacher-clinician [16]. Consequently, it requires the double competencies of a skilled clinician and a skilled teacher. Even though there seem to be many potential candidates for excellent clinical teachers, there are obstacles that effectively prevent them developing expertise in CR. The critical mass of qualified teaching workforce that is needed to make the paradigm change in teaching CR will never accumulate if aspiring young teachers, or those experienced and still willing to learn new didactical methods, do not have time to develop as educators, or if educators treat teaching obligations as a lower priority. Furthermore, if there is insufficient support from leadership and/or there are very limited funds for teaching, people are not likely to be motivated enough to leave their comfort zones to develop skill in this area. Additionally, if there is no culture of giving and receiving feedback on CR performance in a safe and non-judgmental environment, staff may settle for denying their imperfection instead of looking for strategies to improve. This could also be why not much attention is paid to teaching CR theories and strategies for avoiding cognitive biases. Finally, there is also the problem of experienced clinicians’ overly high confidence in their teaching skills. The belief is that if they are qualified enough to have good clinical outcomes, they do not need any guidelines on how to teach CR and, consequently, they do not require any teacher training to hone their CR teaching skills.

Third, after achieving thematic saturation in the interview data analysis, we discussed among ourselves which immediate steps could be taken to most help overcome the identified barrier themes. What seems to us to be the lowest hanging fruit is the theme related to “Lack of awareness about CR”. Plenty of research has been done regarding the development of innovative teaching methods to remedy difficulties in clinical reasoning (e.g. [17, 18]). Teaching aids are available from a variety of sources as free, open-access, medical educational resources: in repositories such as MedEdPortal [19]; on websites of national and international organisations, such as the Society of Improving Diagnosis in Medicine (SIDM) [20, 21]; as outcomes of consensus building initiatives, such as The UK Clinical Reasoning in Medical Education group (CReME) [7]; or as part of web-based CR development tools, e.g. [22]. Despite that, our observation after conducting the interviews is that many of our respondents were either unaware of their existence or were not prepared to use such resources in their teaching practice. To be effective, we believe it is important to go beyond the isolated communities of CR educational experts. We should reach out to those involved in clinical teaching who do not habitually attend medical education conferences or are not interested in research around clinical reasoning. Paradoxically, the current crisis around the COVID-19 outbreak might be an enabling factor. Due to limitations in bedside teaching, many learners and teachers are looking for alternatives or other approaches to supporting their established educational methods [23]. We call for protected time for faculty members to develop their teaching techniques. We should also strive to improve the status of CR teaching by publishing research outcomes that validate the effectiveness of clinical reasoning teaching methods.

The ongoing European project DID-ACT, in the context of which this study was carried out, offers a viable dissemination platform. The longitudinal CR curriculum which is being developed will hopefully help in the translation of explicit clinical reasoning teaching know-how into teaching at many health profession education programs. We believe that it is crucial that clinical reasoning curricula encourage change leaders and advocate the promotion of discussions with colleagues both locally as well as during faculty development workshops or meetings with senior educators. Our own experience so far, and the reaction of some of the respondents, indicates that students might be powerful and enthusiastic allies in this process and may generate additional demand for clinical reasoning teaching abilities.

This study adds to the existing body of knowledge on barriers to the implementation of clinical reasoning teaching [5, 8, 9] and provides a more in-depth analysis of the cultural aspects behind these barriers. For instance, lack of willingness to reflect and learn from errors seems to be a particularly troublesome characteristic of barriers to clinical reasoning teaching that likely impact other curricular barriers. Such observations are not present in previous general-purpose frameworks that were aimed at overcoming inhibitors of curriculum quality (e.g. [24]).

Our observations showed that another example of a curricular barrier that plays an important role but is less emphasized in other frameworks is tensions related to interprofessional aspects. This topic intersects several of the themes identified in our study. We have identified interprofessional tensions not only in communication and collaboration subthemes related to culture, but also in the subtheme regarding how the concept of clinical reasoning is defined. We also think that differences in how health professionals understand and perform clinical reasoning might have influenced some of our respondents’ perceived lack of methods that can be applied to teaching and assessing this competence. It is important to acknowledge that clinical reasoning is not just the domain of one health profession. Different groups should have the right to their own perspectives, and how they think about clinical reasoning should not feel dominated by other professions. It also seems that reconciliation requires an academic (theoretical) and practice-based (intuitive, based on experience) approach to clinical reasoning.

Our study’s strengths include the unique opportunity to involve educators from several European countries. While analyzing the data in the research group meetings, we often expressed surprise concerning how “painfully familiar” the barriers described in one country sounded to those from other countries. However, we also noticed that in some cases, such as standardization of intended learning outcomes and competencies for clinical reasoning and support for teachers’ academic development, some countries (e.g. Germany and Switzerland) seem to be more advanced than other project partners.

Another strength of our research project was that we were not confined to one medical discipline or one health profession but included in our interviews physicians with different specialties, and diverse health professions such as nurses and physiotherapists. We also included participants with varying levels of experience from those who were quite novice through to senior educators or deans.

This study has some limitations. Even though we conducted interviews with representatives from diverse medical and healthcare professions, we reached thematic saturation for them as a group of people connected professionally with the healthcare field, but we did not discuss differences between professions. However, this is the first study that takes into consideration representatives of various healthcare professions and analyzes the collected data together. More research is definitely needed to investigate profoundly the interprofessional context of teaching CR. While we succeeded in including study respondents from several European countries representing diverse cultural backgrounds (which is unusual for this topic), we do not assume that we covered all potential barriers that are characteristic of the whole European context. As the interviews were conducted in many languages, we were reliant on project partners to select the segments and to translate them into English. This study was conducted in the context of the DID-ACT project, in which the authors of this paper are participating. This influenced how the data were collected, which might be seen as a limitation. However, in our opinion the DID-ACT project’s outcomes did not impact the results of our study as the DID-ACT project had just commenced when the research was being carried out. Finally, our study on the barriers to clinical reasoning teaching does not consider any student and patient perspectives’. However, this was a deliberate decision from the start in order to focus on the teachers’ perspective; the reader is referred to other recent studies that explored students’ perspectives on perceived difficulties in clinical reasoning learning [25].

In conclusion, this is an in-depth study exploring the barriers that hinder explicit CR teaching in European institutions and explains the low adoption of longitudinal CR curricula from a broad perspective. In addition to commonly encountered barriers such as lack of teacher time, limited resources, and/or resistance to change, we were able to highlight some that appear to be specific to clinical reasoning. These include lack of culture to reflect on error, tensions due to interprofessional differences, lack of consensus on what CR means, and a variable understanding of the concept of clinical reasoning. In an attempt to illustrate potential causal chains, we also analyzed the connections between the themes. The barriers that hinder CR teaching are complex and should be treated holistically because they likely influence each other in various ways. We suggest immediate follow-up steps to work towards increasing awareness of ways in which clinical reasoning can be taught. The insights collected concerning the complexity and diversity of barriers will help to roll out a long-term agenda to overcome the inhibiting factors in clinical reasoning education, improve the quality of teaching of health professions students, and increase patient safety.

## Supplementary Information

**Additional file 1.** The coding frame.

**Additional file 2.** Interview guide.

## Data Availability

All materials described in this manuscript generated during the current study are available from the corresponding author on reasonable request without breaching participant confidentiality.
