# Supplementary material for: Why is it so difficult to implement a longitudinal clinical reasoning curriculum? A multicenter interview study on the barriers perceived by European health professions educators
Source: BMC Med Educ. 2021 Nov 12;21:575. doi: 10.1186/s12909-021-02960-w (PMC8588939; doi:10.1186/s12909-021-02960-w)
Supplement: Supplementary file 1 — Additional file 1. The coding frame. [file 12909_2021_2960_MOESM1_ESM.docx]

**Appendix 1. The coding frame**

**Table 2. Main themes/subthemes around barriers hindering the adoption of a longitudinal clinical reasoning curriculum**

| **Code** | **Full name** | **Definition** | **Exemplar quotations** |
| --- | --- | --- | --- |
| **Time** | 1. Time-related barriers | Lack of time for: teaching, learning how to teach effectively or teaching CR in curriculum. | |
| for teaching | 1.1. Educators’ lack of time to be teachers | Clinical teachers do not have enough time for any additional teaching obligations. | *"They [teachers] have to deal with a heavy clinical workload which discourages them from doing something new or innovative."(I10)*  *"[...] and they do not have time for that [being taught about thinking] [...] Those teaching students reflection while working on the ward have to be freed from their clinical duties and given time to do that."(I22)*  *“(...) when I talk to students, they sometimes tell me that coronavirus has not made [the quality of] their teaching any worse; in fact, the opposite is true because finally, classes are held, slide sets are prepared... Well, that's weird. So maybe this human resources-related barrier is important after all; the teacher needs to be prepared, he needs to have time, (...) Well, isn't that the same everywhere in medicine?”(I28)* |
| for educators’ development | 1.2.Educators’ lack of time for teacher training | Teachers do not have time to participate in teacher training / they do not have time to invest in learning new methods due to other obligations. | *"... lack of time and clinically engaged physicians (...) difficulty in seeing the benefit. You always have a crude evaluation of the actual benefits of attending courses, sometimes you attend courses in relation to your clinical expertise but could have difficulty in seeing a concrete benefit of this kind of course." (I19)*  *“The hospital is under-financed and staff do not have enough time for anything, but we have a lot of people who are highly motivated. Also, participants have to be able to cancel [their places on] the course [to ensure availability for others] at short notice because of the lack of human resources on their ward. Who will care for patients so that clinicians can be regularly trained in [effectively teaching] CR.”(I20)* |
| in the curriculum | 1.3. Lack of time in the curriculum | The curriculum is already very full and students do not have enough time to participate in more learning activities; the rapid turnaround of patients also hinders the teaching process. | *"There is a battle for time to teach; Once people realize that they may lose time for other things, it may be difficult. If something new is added, something else may no longer be possible."(I3)*  *“Also, these days [clinics are] very rushed; patients arrive and leave on the same day so there’s less time for reflection or for patient assessment.” (I27)*  *“I think it is a problem if clinicians see that [explicit clinical reasoning teaching] clashes with their practical training, e.g. bedside teaching.” (I22)* |
| **Culture** | 2. Culture-related barriers | Culture-related barriers that arise from habitual practices within universities or national health care systems. The lack of: practice in discussing errors, reflection, providing feedback, and intra-/inter-professional communication leads to collaboration issues. | |
| resistance to change | 2.1. Resistance to change | Fear of the unknown. Unwillingness to invest time, to make an effort or to leave comfort zones in order to try something new. Resistance to take on solutions developed outside the given organization (e.g. curricula from abroad)/difficulties with adopting them. | *“Well, as I said, I see a barrier among those, um, senior teachers sometimes; well... maybe it’s their anxiety and fear of something new... [wondering] whether they can handle it.” (I9)*  *"(...) Because yes, because there are several elements: resistance can be caused by fear, resistance can be due to change itself; but also, resistance can arise because different courses and workshops are of a very different quality and people can also be biased to some extent, right? “(I8)*  *"If the curriculum is too general, if the plan isn’t coherent with the [country] context, it could be difficult to practice in universities that lack, for example, physicians. [There are] international differences."(I15)* |
| lack of error culture | 2.4. Lack of culture to reflect on errors | Lack of culture to learn from, analyze and accept errors. People are unwilling to give/receive negative feedback because acknowledging errors and learning from them may undermine their authority. Consequently, junior staff members are afraid to question the behavior of senior staff. Experienced staff members are also unwilling to share their thought processes because this may reveal their errors. | *"If a mistake is made, in order for us to feel good, it is often, much better if we say to ourselves: don't worry it's happened, I don't know... stupid patient or it's an accident at work...(...)...or... or... if I was in your position I would have definitely made the same mistake, right? And somehow... it's not about feeling guilty and punishing yourself, but it is worthwhile looking at something again and saying: ok, what went wrong here? Where was the mistake? instead of looking away, right?" (I8)*  *“Dealing with errors is hard, fear of making errors is very common - a change of culture is needed”(I4)*  *“Our cultural background means that we live in an environment in which we are not criticized by society and everyone may think that individual health professionals are doing good clinical work. Therefore, they do not see the need for this teaching. However, it is quite difficult to know when someone reaches the appropriate ability in CR.”(I25)* |
| lack of self-reflection | 2.5. Lack of culture of self reflection | People do not take time for reflection. There is too much emphasis on taking action without taking the time to consider it. Lack of awareness of the need for continuous improvement in developing one’s competencies. | *“There is probably a belief that once I have learned something I don't need to learn more ... and this [clinical reasoning teaching skills] develops over time.”(I7)*  *“ ...and most importantly, there is a lack of such a skill and... and [a lack of] culture in, uh... asking why [something happened]?”(I8)*  *“[There is an] acceptance issue. I can imagine that experienced clinicians are not really keen on being taught how to split their day between what they usually do and teaching. This can cause uncertainty and many act based on their gut feeling, so some things are done without reflection. It is always uncomfortable to learn that you do not know something.”(I20)* |
| communication issues | 2.6. Difficulties with communication and collaboration around CR intra-/inter-professionally and with patients | Difficulties in communication about clinical reasoning with members of the same profession, inter-professionally, or with patients. A lack of respect for other professions, and dominance of one health profession over another. | *“As far as our university is concerned, the problem [with explicit teaching of clinical reasoning] is that until now only [a faculty member with technical background], who is not a medic, has been involved in it [explicit teaching of clinical reasoning]. It sounded like such an external [artificial] concept. It seems that some theorist just came up with something. And it's like everybody's generally nice and agreeing with each other and generally it's, you know, innovation in the institution, but, you know, the other side of this is that... (...), because, like, it seems to be a different language, with different ways of thinking.”(I29)*  *"It is important to realize that medicine isn’t superior [to nursing or rehab aspects]." (I17)* |
| collaboration between clinics/academia | 2.7. Lack of collaboration between the clinical and academic communities | Tensions between teachers working in clinics and those working in medical and health professions schools (not active, or less active, as healthcare professionals in clinical settings). | *“We are touching on an area where these people (clinicians) feel they are experts. You have to be careful not to offend their sense of expertise, because that's just... well, that's the end (of collaboration)”(I18)*  *"Who will put their name against this type of CR course? If such a course is led by someone non-medical, it will not be taken seriously; but if it is led by one of the department heads, it may not be accepted by other departments or other department heads. This is a political issue.”(I20)*  *"(...) There are academics [nurses] who indicate that they [clinical nurses] feel they have no status and cannot do anything. ... So, there are those of us who have a problem with that."(I24)* |
| **Motivation** | 3. Lack of motivation/incentive to introduce the curriculum | A theme that includes lack of funding (resources to pay teachers, cost to organize courses) or other forms of motivation or incentive (lack of support from authorities, low-priority for teaching). | |
| lack of financial incentive | 3.1. Lack of financial resources | This barrier is caused by the lack of financial resources to pay teachers, organize courses, and buy training resources, as well as lack of clarity on who will fund courses. | *“Teaching takes time, and a university is there to teach. No, it shouldn't be a financial issue, but it is. You have to provide a financial incentive for people so that they're [able to take] responsibility for teaching during working hours”(I10)*  *“It could be that people have different expectations to improve their teaching. Someone is, let's say, a clinician, but he has a simple problem: he has a private practice in the afternoons. Maybe you will have to pay him so he is keen to share his knowledge. This is an extreme example.” (I28)*  *“I see a challenge in the lack of resources. You need money for the administrative staff [to organize] trainer training tutorials.”(I22)* |
| lack of top-down support | 3.2. Lack of support from the top (higher positions/institutions) | Lack of support from the top (e.g. team leader, dean, ministry of health, etc.) due to several reasons (lack of positive stimulation or encouragement) and administrative obligations. | *"...lack of conviction that this is important among decision makers […] and this is quite important, because in order to have support in implementing something new, we must have support not only from the bottom-up, but also among those who make decisions at the top."(I7)*  *“(...)it is also forced by the [public] payer. ...a nosological approach is being pushed, which means that... umm... it's necessary to diagnose disease in order to get funding, for example…”(I8)* |
| teaching priority | 3.3. Teaching as low(er) priority | Clinical or research activities are regarded as more important than teaching activities. Low-esteem within teaching in general. | *" »There's nothing more stupid than teaching students.« That was an original statement by a colleague” (I10)*  *“We have a lack of time [as we have to] stretch ourselves between clinics and education” (I25)* |
| other | 3.x. Other motivational aspects | Lack of adequate promotion/dissemination of courses. | *"People have to be made aware that this course exists. It has to be communicated so that the target group receives the information." (I1)* |
| **Concept** | 4. Understanding of the clinical reasoning concept | Lack of awareness of the importance of CR and disagreement over what it means for a profession or interprofessionally. | |
| awareness of importance | 4.1. Lack of awareness of clinical reasoning and its importance | Medical knowledge is perceived as sufficient for responders to be good clinicians and good teachers. The teaching of CR is perceived as not important. | *"98% of the teachers do not know anything about CR." (I6)*  *“The barriers are more psychological, but maybe they are not even psychological. Simply put, we haven't heard of it [CR] before. Everyone does it, even subconsciously, but the name ["clinical reasoning"] itself … Think about that name”(I28)*  *“But the point is also that, in order to study, teachers themselves, as well as students, (...) must know that it [CR] is important... I'm sorry, it should be good to have a challenge and for them to become competent in it [CR]. Exactly the same thing has to happen for teachers: they have to feel competent, they have to feel that it's important, and it has to be interesting and challenging for them.” (I8)* |
| semantics | 4.2. Lack of agreement what clinical reasoning is | People disagree what clinical reasoning is; there is no one simple definition. This includes lack of agreement between professions on what constitutes clinical reasoning. | *“[It is] important to make CR more concrete.  It [CR] is complex and it is important to continue [investigating] working with it. (I18)*  *"Clinical reasoning is not only about identifying and treating something, but it is much more. Like the bio-psycho-social model. We always talk about patients but we also have healthy people who come for check-ups, vaccinations, or whatever. A holistic approach for all patient groups including all religions, languages, regions, disabled persons, men/women/kids (...) I would like to see the human being as the focus. The term clinical reasoning is misleading, because the term 'human being' is not present, it sounds very technical. And I also miss the ambulatory context." (I20)*  *“Also, there needs to be a standardized national concept for this [how to teach CR]. Besides that, I don't see any issues. But, for sure, it depends on the actual concept of the curriculum.”(I2)* |
| **Teaching** | 5. Teaching process of clinical reasoning | A theme that includes lack of awareness or belief in the effectiveness of explicit CR teaching methods; lack of CR competency frameworks; guidelines to teach CR;  lack of adequately trained instructors, lack of leadership in both implementing and running CR courses. | |
| disbelief in explicit CR teaching | 5.1. Belief that CT cannot be taught at all, or is not effective when taught explicitly | A belief that clinical reasoning comes with age and experience; people are convinced there is no point in formally teaching it as this is ineffective. | *"There are people who without learning CR are somehow perfectly able to reason and they can say: after all, I've learned to reason very well, I'm great at it, so why do they need to come here?"(I29)*  *“Sadly, some colleagues do not think it is obvious/natural to have students and to treat them as apprentices. The profession is still one that very much relates to apprenticeships.” (I11)*  *“All over the world, since the very beginning, there have been two barriers to introducing the teaching of communication skills: 1. communication cannot be taught - this is something that somebody is born with; 2. the only form of teaching is experience (this respondent has been involved in the implementation of a communication course at [a university] since it started). It seems to me that these are similar barriers. Similar to some kind of thinking schema”(I29)* |
| lack awareness of methods | 5.2. Lack of awareness that clinical reasoning can be taught | Teachers recognize problems with clinical reasoning but are not aware of ways to resolve them. People are unaware how to teach CR, but react positively to information that it can be taught and are interested in exploring the efficacy of those methods. | *“If you look at who attended the DID-ACT kick-off meeting, you will see that there is not much respect for this topic. I don't know why this is; maybe they do not know that you can learn clinical reasoning, which is a modern term for clinical practice and expertise. “(I22)*  *“There are teaching assistants who show their ways of thinking, share their own diagnostic path, so to speak, and illustrate this process, which, for example, gives students some insight into the methodology of developing a diagnosis. However, some clinicians are not even aware of this process and somehow... Because there is a difference between doing something and teaching it, right?” (I8)*  *"Most people don't know how to teach clinical reasoning. There is ignorance. What you don't know, you can't implement."(I10)* |
| lack of competency frameworks | 5.3. Lack of official learning objectives (frameworks) | Perceived lack of competency frameworks or other teaching standards that would justify teaching clinical reasoning. | *"Our curriculum is based on learning objectives that are provided to us by the ministry […] I'm not sure, but it seems to me that there is no clinical reasoning there at all."(I7)*  *“(...)a very traditional domain-oriented curriculum with fixed responsibilities; [it is] very hard to integrate a new/longitudinal aspect”(I4)* |
| no specific guidance | 5.4. Lack of guidance on how to teach | Lack of awareness of detailed instruction on how to apply specific clinical reasoning teaching methods and for running teacher training on clinical reasoning teaching. | *“Additionally, there are no guidelines on how to teach CR to teachers and how to create a curriculum. Other obstacles, such as a lack of trained individuals, can be overcome, but most important is the mentality and direction behind doing it [CR teaching].”(I26)*  *"...I realized then that just because someone can communicate well, it doesn't mean they can teach it [CR]. It could be the same with clinical reasoning, right? If you don't have it clearly laid out in your mind, you can't really teach it. If you don't have this type of clear method, [you can’t teach CR]. (...) Not every great player is a good coach." (I29)*  *“We need guidelines, recommendations for [teaching] CR: recommendations would enhance our understanding. So what standards are required to take a clinical history, what are the guidelines for teaching clinical skills? And how do we measure the outcomes? Is this something that will help us to diagnose heart murmurs earlier? This is probably part of the [aims of the] DID-ACT project: to unify and create a consensus of the evidence required to conduct CR teaching”(I25)* |
| lack of qualified educators | 5.5. Lack of qualified educators | People are not qualified enough to teach clinical reasoning or conduct clinical reasoning teacher training. | *“Teachers do not have enough teacher training. If you are a doctor, you are not taught how to teach and [are not taught] what [teaching] methods are available.”(I10)*  *“It is easier to find clinicians for clinical skills training rather than tutors, even if the tutor has far less to do because students do all the work”(I22)*  *“(...) feeling that colleagues are quite well-versed in information technology, especially young colleagues. They like evidence-based, graphics-based material. They use the material well [in their teaching]. So, we need additional training in the CR construct. Most colleagues, including me, do not have a theoretical background in CR. If we had that, our teaching would probably be better." (I25)* |
| lack of leadership | 5.6 Lack of leadership in longitudinal curriculum implementation | Perceived lack of which person or organizational unit is responsible for implementing a longitudinal clinical reasoning curriculum. | *“We believe we have or strive for through progression within the programs, the challenge is that many are involved and it easily becomes cluttered and unclear who has the mandate to actually take control and make decisions. That makes it hard, but I don't see how it could be any different.” (I14)*  *“It will not work to just have the course: rolling it out is crucial, and this rollout cannot be generalized; a tailored approach is needed” (I6)*  *“[By] replacing colleagues and managers [there is a danger that] the history and reasons for including certain things [in the curriculum] information, etc. will disappear. Within an unstructured organization you don’t have a specific assignment to deliver, and there will be different changes [to the curriculum] all the time. Continuity is difficult to maintain.”(I13)* |
| other | 5.x. Teaching process - other | For instance, lack of acceptance of clinical reasoning teaching methods. | *“(...) also, students have to be trained from the start in new/innovative teaching methods, so a mixture of teaching methods is required from the first year onwards”(I4)* |
| **Assessment** | 6. Assessment of clinical reasoning | Unawareness or inability to implement clinical reasoning assessment methods. | |
| awareness or implementation issues | 6.1. Unawareness of good assessment methods or difficulties in implementing them | Lack of (or unawareness of) clinical reasoning assessment methods or the preference (more trust in) for traditional assessment methods. | *"How do you know how good you are or when you have learnt enough?" (I18)*  *“The challenge is that it’s a skill that you can never learn well enough. Students get frustrated  because of this. It’s hard to find any frame of reference [for assessments] since we lack [documented] factual knowledge.”(I18)*  *“Currently, there are not many assessment methods because traditional formats are preferred, such as MC questions and also OSCEs of varying quality”(I20)* |
|  |  |  |  |
| **Infrastructure** | 7. Infrastructure and logistics | Difficulties in organizing clinical reasoning teaching and assessment due to lack of physical space, adequate hardware/software infrastructure, and workflows. | |
| physical learning space or logistics | 7.1. Small rooms, large groups, or lack of clerkship places for students | Lack of or insufficiently large classrooms, groups that are too large for teaching clinical reasoning, and lack of clerkship places for clinical teaching. | *"To maximize the learning outcome, the group size, technical infrastructure and room availability are relevant, but these are limited. It is trivial but still important." (I23)*  “*We don't have many hospital places and have not really learnt how to involve students in outpatient work, which is now a big part [of health care services].*“ (I11)  *“I think group size could also be a barrier... Learning clinical reasoning in a group of 15 people can be difficult." (I28)* |
| software | 7.2. Lack in IT infrastructure | Lack of IT tools, or the current IT tools do not meet all expectations. | *“[The VP tool] is not attractive to me. It might be silly, but there are some educational platforms which I am happy to use again; [our current VP platform] has something about it that is not appealing to me and I feel that it's not just my view… when you talk to students, they agree that the VP tool has its disadvantages. I don't know if it's about aesthetics, but you know, while something like that [the VP tool]... has many advantages, it also can have disadvantages as a result of those advantages. It has the advantages of simplicity, accessibility, ... but, uh, it is what it is ... well, just, uh, I'm not enjoying using it.” (I29)* |
| **Others** | **8. Others** | A residual theme containing segments we could not assign otherwise. | |
|  | 8.1. No barriers, unclear or evasive responses | Perception that there are no barriers to introducing clinical reasoning teaching which sometimes leaves the impression there is a latent reason that inhibits implementation of the CR curriculum. | *“Basically, I don't see any barriers; as long as it [CR teaching] does not come with unrealistic demands, it is something that can be aligned with the block of internships.”(I2)*  *“Well, firstly, I don't think the students feel there are any barriers. They're probably keen on being taught clinical reasoning, I hope so. (laughs)”(*I9*)*  *“If there is a systematic approach, students will probably accept this positively.”(I20)* |
