# Supplementary material for: Why is it so difficult to implement a longitudinal clinical reasoning curriculum? A multicenter interview study on the barriers perceived by European health professions educators
Source: BMC Med Educ. 2021 Nov 12;21:575. doi: 10.1186/s12909-021-02960-w (PMC8588939; doi:10.1186/s12909-021-02960-w)
Supplement: Supplementary file 2 — Additional file 2. Interview guide. [file 12909_2021_2960_MOESM2_ESM.docx]

# **DID-ACT Project: Specific Needs Analysis- Barriers**

**Interview guide version: Teachers/Faculty Barriers**

**General interview rules:**

- **The order of the questions can change with the “natural flow” of the interview. However, make sure that you ask all the questions.**
- **You can ask the questions using your own words, but the meaning should not change.**
- **Unless it is not necessary (e.g. demographics), ask open questions that cannot be answered with a simple yes or no whenever possible.**
- **If an answer has been vague or superficial, ask for more details. Our goal is to receive in-depth information and understanding that can be used in the curriculum development process.**
- **Before starting the interview, let the participants sign the declaration of consent.**
- **Don’t forget to start the audio recording after you received the participants’ consent.**
- **If possible, take notes of the answers (short notes)**

***Note for the interviewer: Describe the content of the text below***

Clinical reasoning encompasses health professionals’ thinking and acting in assessment, diagnostic and management processes in clinical situations. Despite the importance of clinical reasoning, there is still a lack of explicit teaching and learning activities of clinical reasoning.

To fill this gap, the EU project DID-ACT ([https://www.did-act.eu](http://www.did-act.eu)) is planning to develop a students' and train-the-trainer curriculum on clinical reasoning.

In this survey we will ask you questions about how clinical reasoning is and should be taught and assessed at your institution, as well as what a train-the-trainer course should ideally look like. Your input is highly valued and will help to design the DID-ACT curricula.

Please answer the questions as accurately as possible. Your answers will be kept confidential.

The findings from this survey are also going to be published in congress contributions and scientific publications. With your participation you agree to the use of your data for these purposes.

***Note for the interviewer: Let the participant sign the declaration of consent now. Start the audio recording afterwards, before you start with the actual interview.***

**1. In which country do you work?**

**2. In which institution do you work?**

**3. What educational programme do you relate mostly to**Medicine

- Nursing
- Physiotherapy
- Occupational therapy
- Other (please specify)

**4. How would you describe your primary role/roles at your institution?**

Healthcare Professions Educator

- Physician
- Nurse
- Physiotherapist
- Occupational therapist
- Researcher
- Dean
- Curriculum Planner/Manager
- Course Director
- Student
- Other (please specify) …

**5a. How many years of experience in healthcare education (excluding years of study) do you have?**

**6. How much experience do you have regarding clinical reasoning in:**

- Teaching
- Assessment
- Faculty development
- Clinical practice

**7. How do you personally understand CR?**

**Part B Students Curriculum**

In the EU project DID-ACT it is planned to develop a modular, adaptable longitudinal students-curriculum on clinical reasoning which is based on a variety of methods including virtual patients and blended learning, as well as assessment.

In the following, we will ask you questions about the student curriculum.

**Part C Barriers/solutions for teaching and assessment of clinical reasoning**

**8. What, in your opinion, are the main barriers/ challenges for introducing such a clinical reasoning curriculum for students at your institution?**

***Note for the interviewer: Please ask this question open first. Then hand the list out to the participants. Go through the list together and note the answers to aspects that have not been mentioned already.***

- No particular challenges
- Lack of qualified faculty to teach clinical reasoning
- Lack of curricular time
- Lack of financial resources
- Lack of guidelines for clinical reasoning curriculum development
- Lack of awareness of the need for explicit clinical reasoning teaching
- Lack of top-down support
- Perception that clinical reasoning cannot be taught
- Curriculum invented elsewhere
- Don't know
- Other (please specify)

**9. Do you have a train-the-trainer course on clinical reasoning at your institution? If yes, please describe.**

**10. Do you think the DID-ACT train-the-trainer course is necessary for healthcare educators at your institution? Why yes / why no?**

**11. What should the DID-ACT train-the-trainer course on clinical reasoning cover?**

***Note for the interviewer: Please ask only the open question- we concentrate on barriers but it is good to have common understanding of what the TTT should contain.***

**Part C Barriers / Solutions**

**12. What critical aspects/barriers/challenges do you see in implementing the DID-ACT train-the-trainer course at your institution?**

***Note for the interviewer: Please ask this question open first. Then hand the list out to the participants. Go through the list together and note the answers to aspects that have not been mentioned already.***

- No particular challenges
- Lack of qualified trainers to teach the train-the-trainer course
- Lack of time of trainers
- Lack of time of participants
- Lack of financial resources
- Lack of guidelines for teaching and assessing clinical reasoning
- Lack of awareness of the need for a train-the-trainer course
- Lack of top-down support
- Perception that clinical reasoning cannot be taught
- Course invented elsewhere
- Don't know
- Other (please specify)

**13. What incentive other than a certificate might be helpful for motivating participation in this course?**

**Part D Final question of the survey/interview**

**14. Do you have any further comments?**
